# Ligneous Periodontitis in a Patient with Type 1 Plasminogen Deficiency: A Case Report and Review of the Literature

**DOI:** 10.1155/2020/5680535

**Published:** 2020-03-24

**Authors:** Arun Sadasivan, Roshni Ramesh, Deepu George Mathew

**Affiliations:** ^1^Department of Periodontics, Sree Mookambika Institute of Dental Sciences, Kulashekaram, Tamil Nadu, India; ^2^Department of Periodontics, Government Dental College, Thrissur, Kerala, India; ^3^Department of Oral and Maxillofacial Pathology, Annoor Dental College, Muvattupuzha, Kerala, India

## Abstract

**Background:**

Ligneous periodontitis or destructive membranous periodontal disease is a rare condition involving gingival tissues, which is due to plasminogen deficiency and fibrin deposition. Plasminogen deficiency is an ultrarare autosomal recessive disease. The disease is characterized by gingival enlargement and periodontal tissue destruction that leads to rapid tooth loss despite treatment attempts. A defect in fibrinolysis and abnormal wound healing are the main pathogenesis of this condition. It is caused by mutations in *PLG*, the gene coding for plasminogen, which results in decreased levels and functional activity. *Case Presentation*. In this case report, clinical and histopathological findings of a 26-year-old male patient who presented with generalized membranous gingival enlargement are presented. He was the third child of consanguineous parents and had multicystic congenital hydrocephalus at birth. Besides the gingival enlargement, he also presented ligneous conjunctivitis since childhood. The intraoral examination revealed generalized periodontal breakdown. Radiographs showed alveolar bone loss present in every quadrant. All blood investigations were normal except for plasminogen deficiency. A biopsy sample was excised from affected gingiva and a series of histopathological evaluation was performed. Based on clinical and histopathological evidence, a diagnosis of destructive membranous periodontal disease or ligneous periodontitis was made. A clinical exome assay for the PLG gene was also done. It was confirmed as Type 1 plasminogen deficiency.

**Conclusion:**

Ligneous periodontitis has been rarely reported in India. The reasons could be because of the rarity of the disease or missed diagnosis. The need to take a proper history and perform a proper clinical examination and histopathologic evaluation has to be stressed when diagnosing and treating gingival enlargements. If a genetic condition is suspected, genetic screening is also needed. All these will help the clinician in correctly diagnosing the disease and formulating a proper treatment plan for managing the condition.

## 1. Introduction

Gingival enlargements are a common feature seen in patients which present aesthetic, functional, psychological, and periodontal problems. The etiology in most of the cases has an inflammatory, hormonal, drug-induced, idiopathic, or cancerous origin. There are, however, certain rare conditions in which the etiology is obscure. One such condition is destructive membranous periodontal disease or ligneous periodontitis (LP). This condition has been attributed to a plasminogen deficiency. Other possible causes such as autoimmune or hypersensitivity reactions, trauma, and viral or bacterial infections have been reported [1, 2].

Haemostasis depends on many factors like vasoconstriction, formation of blood coagulation factors, and fibrinolysins, especially plasmin. Plasmin is derived from plasminogen (PLG), which is its proenzyme; it is synthesized in the liver and circulates in the plasma. PLG plays an important role in intravascular and extravascular fibrinolysis and wound healing. It is converted to plasmin by cleavage of the Arg561-Val562 peptide bond by either a tissue-type PLG activator (tPA) or a urokinase-type PLG activator (uPA). Activation of PLG by tPA is the major pathway that leads to efficient lysis of fibrin clots in the blood stream, whereas activation of PLG by uPA seems to be mainly responsible for mediating PLG activation in association with cell surfaces especially wound healing and tissue remodelling [3]. Plasmin also acts as a broad spectrum proteolytic factor either directly by degrading extracellular matrix proteins, e.g., laminin, fibronectin, and proteoglycans, or indirectly by activating latent metalloproteinases. Therefore, it plays a crucial role in tissue homeostasis, e.g., remodelling, angiogenesis, and wound healing. Moreover, plasmin has also been found to play an important role in host defence against infections [4, 5].

Plasminogen deficiency is a rare (1.6 in 1 million individuals) autosomal recessive disease caused by homozygote or compound-heterozygote mutations of the plasminogen gene PLG. The PLG gene maps to chromosome 6q26-q27. It spans about 52.5 kilobases (kb) of DNA and consists of 19 exons and 18 introns. The PLG cDNA of 2.7 kb encodes a protein consisting of 791 amino acid residues [6]. There are two types of plasminogen deficiency: (1) hypoplasminogenemia (Type I PLG deficiency), in which there is a marked decrease in the levels of PLG antigen ≤ 1.9 mg/dL (normal range 6 to 25 mg/dL) and functional activity of up to 33% (normal range 80 to 120%), and (2) dysplasminogenemia (Type II PLG deficiency), in which the level of immunoreactive PLG is within normal range but the specific activity of PLG is reduced [7–10].

Gingival enlargement in hypoplasminogenemia has been described as “amyloidaceous ulcerated gingival hyperplasia” or destructive membranous periodontal disease (ligneous periodontitis). The term ligneous periodontitis (LP) was first coined by Gunhan et al. to describe a destructive membranous periodontal disease [11]. LP generally starts in childhood with conjunctiva as well as gingival mucosa as its most affected sites [1, 12]. Ligneous periodontitis is characterized by gingival enlargement and severe attachment loss, which is associated with plasminogen deficiency, the accumulation of amyloid-like material in the lamina propria, and deposition of fibrin [5, 11, 13]. Most cases of LP have been reported in association with ligneous conjunctivitis (LC), which suggests that both clinical manifestations may be related. LC is a rare form of chronic conjunctivitis that usually affects children, girls more often than boys (3 : 1), and can occur at any age. It is characterized by fibrin-rich pseudomembranes mainly on tarsal conjunctivae [1, 8]. Type II PLG deficiency patients, however, have never reported developing pseudomembranous lesions.

In this case report, the clinical, histopathological, and genetic evaluation of a 26-year-old male patient who presented with generalized membranous gingival enlargement is presented.

## 2. Case Presentation

A 26-year-old male patient reported to our clinic with complaints of generalized swelling of gums and pain and bleeding from gums. Gingival enlargement was present since childhood and was slowly increasing in size. The enlargements had been previously localized in sites and had been surgically excised at 8 years of age, but recurrence occurred. He had periodic oral prophylaxis done. He was the third child of second degree consanguineous parents. At birth, he presented with multicystic hydrocephalus which was drained by ventriculoperitoneal shunt. Since an early age, he also presented with profuse granulomatous growth in conjunctiva in both eyes. Corneal involvement and chronic obstruction led to the progressive blindness of the left eye by the age of 15. Histopathological evaluations led to a diagnosis of ligneous conjunctivitis.

### 2.1. Clinical and Radiographic Examination

**Figure 1:**
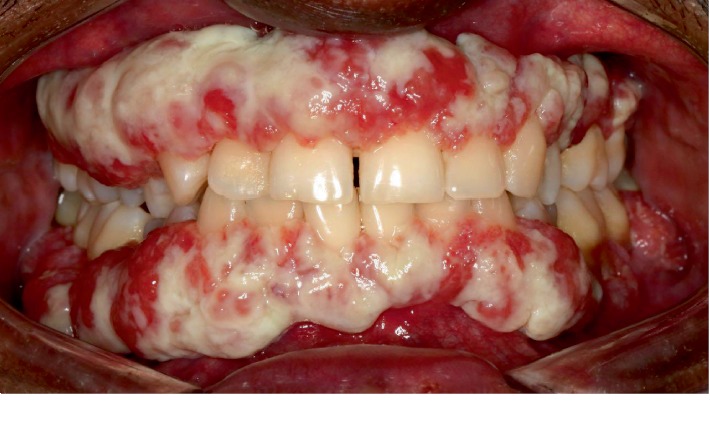
Intraoral examination of a 26-year-old male patient with PLG deficiency (Type 1) showing membranous gingival enlargement.

**Figure 2:**
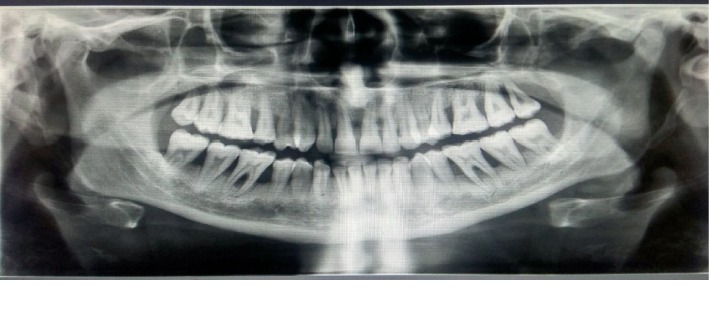
Panoramic radiograph showing extensive generalized periodontal breakdown.

**Figure 3:**
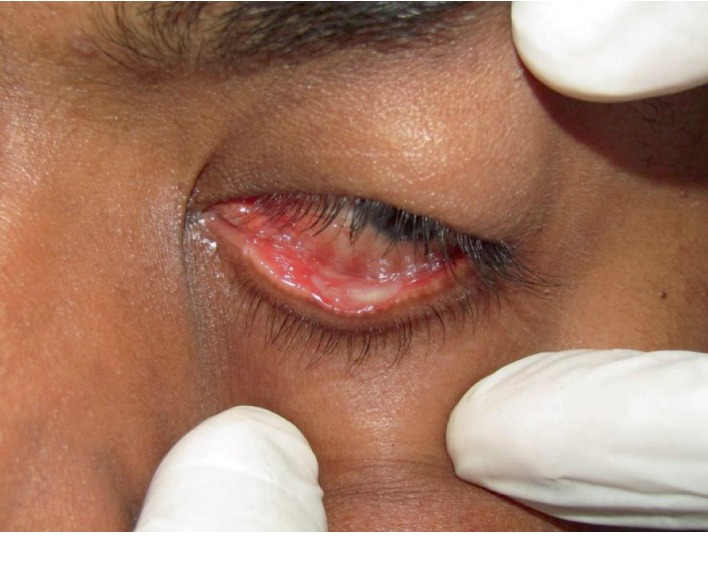
Ligneous conjunctivitis.

The intraoral examination revealed solid, nodular, and fragile erythematous and hyperplastic gingival enlargements involving the marginal and attached gingiva in both maxillary and mandibular arches. The periodontal evaluation revealed signs of generalized severe periodontitis, ulceration, and white-yellow fibrinous pseudomembranes (Figure 1). Probing showed increased pocket depths ranging from 8 to 10 mm in many teeth, bleeding on probing in over 30% of sites, and generalized tooth mobility. The panoramic radiograph showed generalized alveolar bone loss of more than 50%, especially in the posterior teeth (Figure 2). Recurrent conjunctivitis was seen as a white membrane in the mucosa of the upper and lower eye lids (Figure 3). The patient's growth and development were within normal limits, and no other family member reported similar findings.

### 2.2. Laboratory Investigations

Haematological assessment and biochemical tests were done all of which were within normal parameters except for plasma plasminogen activity which showed a level of <5% (biological reference=low, reference interval: 75‐140%), a lactate dehydrogenase (LDH) level of 331 IU/L (reference range = 135‐214), SGOT/AST levels of 60 IU/L (reference range = 15‐37), and SGPT/ALT levels of 66 IU/L (reference range = 30‐65). Plasminogen deficiency was diagnosed as due to hypoplasminogenemia which is named Type I PLG plasminogen deficiency. Haematological evaluation and biochemical tests such as liver function tests, serum proteins, azotemia, glycemia, hormonal values, and kidney function tests were within normal limits.

### 2.3. Histopathological Examination

**Figure 4:**
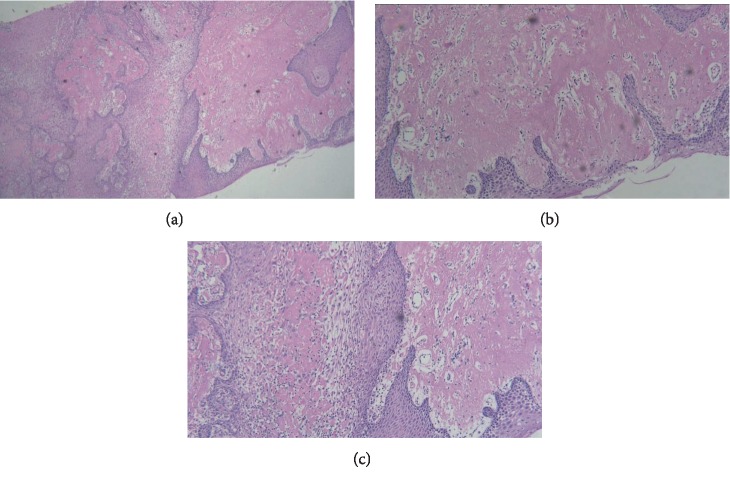
(a) H&E stain; original magnification is ×4, while magnification in (b) is ×10 and magnification in (c) is ×40. Photomicrograph showing parakeratinised stratified squamous surface epithelium with branching and anastomosing rete ridges and connective tissue showing subepithelial deposits of homogenous eosinophilic material that resembled amyloid. The deeper parts show a diffuse mixed inflammatory cell infiltrate comprising predominantly of neutrophils.

Under the effect of local anaesthesia, an incisional biopsy of the gingival enlargement was done. The haematoxylin-and-eosin-stained soft tissue section showed a parakeratinised stratified squamous surface epithelium with branching and anastomosing rete ridges in association with a fibrovascular connective tissue. The connective tissue showed subepithelial deposits of homogenous eosinophilic material that resembled amyloid. These areas were relatively acellular. The deeper parts of the connective tissue showed a diffuse mixed inflammatory cell infiltrate comprised predominantly of neutrophils, lymphocytes, and plasma cells. Sections also showed capillary vessels and extravasated red blood cells. Gingival biopsies all stained for fibrin but stained (Congo Red) negatively for amyloid, lipid, keratin, immunoglobulins, and glycogen. Masson's trichrome was negative and PAS variably positive (Figure 4).

### 2.4. Genetic Testing and Variant Interpretation

**Figure 5:**
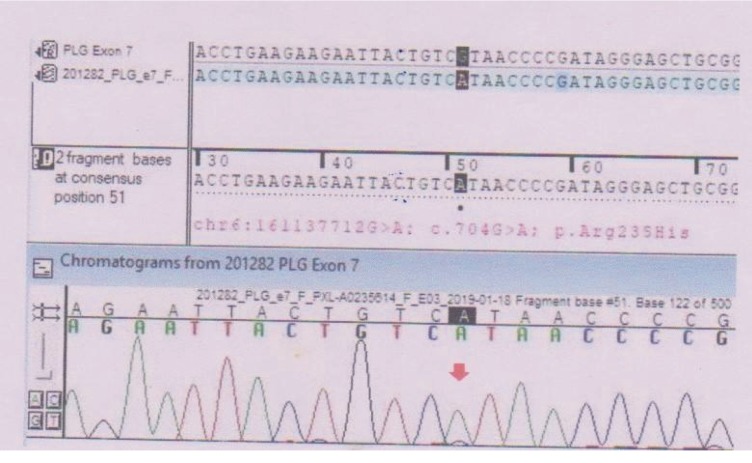
Sequence chromatogram and alignment to the reference sequence showing the variation in exon 7of the PLG gene (chr6:161137712G>A; c.704G>A; p.Arg235His) detected in homozygous condition in the patient.

After the clinical and histopathological diagnosis of ligneous periodontitis associated with Type 1 plasminogen deficiency was made, DNA tests were done to the patient at MedGenome laboratories, Bangalore, India. The test methodology performed was targeted gene sequencing. Selective capture and sequencing of the protein coding regions of the genome/genes was performed. Mutations identified in the exonic regions are generally actionable when compared to variations that occur in noncoding regions. Targeted sequencing represents a cost-effective approach to detect variants present in multiple/large genes in an individual. DNA extracted from blood was used to perform targeted gene capture using a custom capture kit. The libraries were sequenced to mean > 80‐100x coverage on an Illumina sequencing platform. The sequences obtained are aligned to a human reference genome (GRCh37/hg19) using the BWA program [14, 15]. It was analyzed using Picard and GATK version 3.6 to identify variants relevant to the clinical condition [16, 17] (Figure 5).

Variant interpretation was a homozygous missense variation in exon 7 of the PLG gene (chr6:161137712G>A; depth: 50x) that results in the amino acid substitution of histidine for arginine at codon 235 (p.Arg23His; ENST00000308192.9). Inheritance was autosomal recessive. The observed variation lies in the kringle domain of the plasminogen protein [18] and has previously been reported as pathogenic as per ClinVar and SwissVar [19, 20]. The p.Arg235His variant has not been reported in the 1000 Genomes, ExAC, and the internal database of the MedGenome laboratories. The in silico predictions of the variant are probably damaging by PolyPhen-2 (HumDiv) and damaging by SIFT and MutationTaster 2. The reference codon is conserved across species. For the OMIM phenotype, Type 1 plasminogen deficiency (OMIM#217090) is caused by homozygous or compound heterozygous mutations in the PLG gene (OMIM∗173350).

### 2.5. Treatment Done

**Figure 6:**
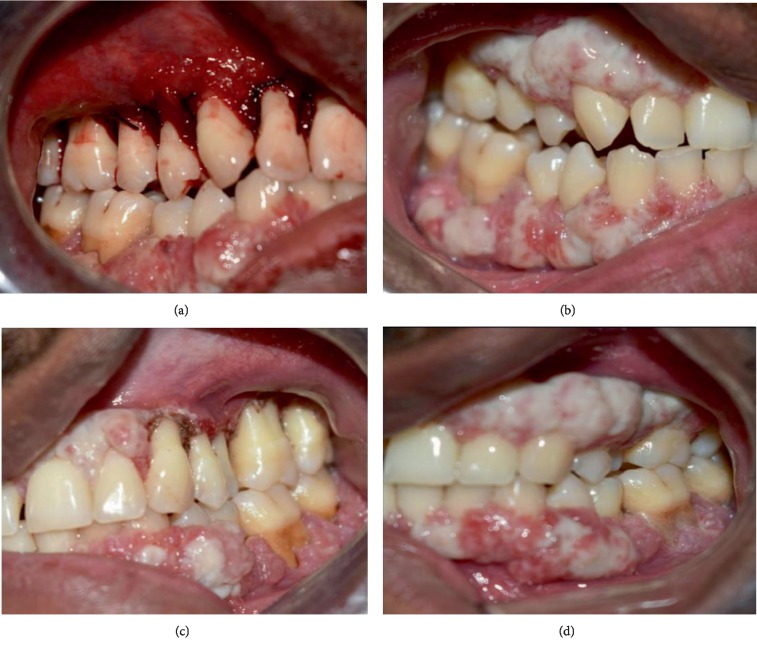
(a) Surgical excision by internal bevel gingivectomy on the right maxillary region. (b) 3-Month follow-up shows recurrence of the lesion. (c) Excision by diode laser on left maxillary region. (d) 3-Month follow-up shows recurrence of the lesion.

After the diagnosis of LP, the management of the dental condition was done. Supra- and subgingival debridement was performed under local anaesthesia. Oral hygiene instructions were given to the patient who was advised to rinse twice daily with 0.2% chlorhexidine digluconate for a period of two weeks. A marked reduction in pocket depth and bleeding on probing was noticed after eight weeks, but the enlargements still persisted. Surgical excision of the lesion was done by internal bevel gingivectomy on the upper right quadrant and by a diode laser in the upper left quadrant. However, recurrence of the lesion was seen after 3 months (Figure 6). The patient was also referred to an ophthalmology clinic for the management of ligneous conjunctivitis.

## 3. Discussion

In this report, a case of LP has been presented along with its corresponding clinical, radiographic, histopathologic, and genetic findings in addition to its management by excision. Only a limited number of cases with plasminogen deficiency and oral lesions have been reported in the literature [5]. Congenital plasminogen deficiency is a rare autosomal recessive disease which is clinically characterized by chronic mucosal pseudomembranous, nodular enlargements in both the maxilla and mandible depending on the subepithelial fibrin deposition and inflammation [11, 21]. It is now well documented that hypoplasminogenemia is a multisystemic disease. Clinical manifestations in a collection of 74 patients with severe (i.e., homozygous or compound-heterozygous) hypoplasminogenemia showed a median age of first clinical manifestation of 9.54 months (range 3 days to 61 years). The female-to-male ratio was 1.43 : 1. The majority of the affected patients suffered from ligneous conjunctivitis (81%) and ligneous gingivitis (30%). Other manifestations included the involvement of the upper and lower respiratory tract (20%), the middle ear (15%), the female genital tract (9%), the gastrointestinal tract (2.7%), the kidneys (4%), and the skin (juvenile colloid milium: 1%). Congenital occlusive hydrocephalus was seen in 12% of the patients. A few (5%) patients exhibited Dandy-Walker malformation, a congenital hypoplasia, and upward rotation of the cerebellar vermis and cystic dilation of the fourth ventricle [7, 22–25]. In the present case, the patient presented with congenital hydrocephalus, ligneous conjunctivitis, and enlargements in both jaws since childhood. Based on the clinical and radiological findings, a provisional diagnosis of LP was considered.

The exact pathophysiology of the clinical presentation seen in LP is still not clear. In vitro data and animal studies indicated that alterations in tissue repair and host defense mechanisms are responsible for the onset and the progression of periodontal destruction [4, 26]. Plasminogen plays an important role in intravascular and extravascular fibrinolysis, wound healing, cell migration, tissue remodelling, angiogenesis, and embryogenesis [27]. Local extracellular fibrinolysis by plasmin is required for the initial removal of the fibrin-rich matrix as well as for the remodelling of the granulation tissue and completion of wound healing [10, 28, 29]. Impairment of the pathway due to hypoplasminogenemia leads to fibrin accumulation and an increased inflammatory reaction. Consequently, the process of wound healing stops at the stage of granulation tissue formation and cellular proteolysis, which may then further support the invasion of pathogens. This process is notably pronounced in mucous membranes such as the periodontal tissues [29, 30]. The fact that only 32% of patients who suffer from PLG Type 1 deficiency develop ligneous periodontitis strongly supports the notion that external triggers, i.e., trauma or infection, may play an additional significant role in the pathogenesis of this disease [5, 10, 31]. The average age of patients reported in the literature with ligneous periodontitis is 12-18 years, while isolated cases have been reported in older patients; these differences may reflect variability in PLG activity due to different plasminogen gene mutations [32].

Histopathological examination was done to confirm the diagnosis. Severe acute inflammation and irregular acanthosis of the epithelium with extensive fibrin leakage and the accumulation of an amyloid-like material in the lamina propria is the characteristic feature of this disease at histopathological examination. This condensed fibrin accumulation lacks reticulin fibres [11]. A variety of other hyaline deposits, ranging from classical amyloid to mixtures of immunoglobulins, fibrin, fibrinogen, and albumin, might be found in the gingiva. Indeed, amyloid is itself a nonspecific term for a spectrum of entities, including immunoglobulin light chains, serum amyloid fibrillar proteins, *β*2-microglobulin, keratin transthyretin, and others [33]. In the present case too, the section showed parakeratinised stratified squamous surface epithelium with branching and anastomosing rete ridges in association with a fibrovascular connective tissue. The connective tissue showed subepithelial deposits of homogenous eosinophilic material that resembled amyloid. These areas were relatively acellular. Gingival biopsies all stained for fibrin but stained (Congo Red) negatively for amyloid, lipid, keratin, immunoglobulins, and glycogen. Masson's trichrome was negative and PAS variably positive. These findings confirmed the diagnosis of LP.

Inherited homozygous or compound-heterozygous hypoplasminogenemia is a very rare disorder (with an incidence estimated at below 1 : 10) which is associated with deficient extravascular PLG-dependent fibrinogen clearance leading to impaired wound healing mainly of mucous membranes. In affected patients, the wound healing capacity seems to be arrested at the stage of granulation tissue formation [8]. Studies suggest that the K19E mutation is the most common molecular genetic defect in patients with hypoplasminogenemia worldwide [25]. A variety of other genetic abnormalities have been identified in the PLG gene in subjects with heterozygous, homozygous, and compound-heterozygous hypoplasminogenemia: missense mutations include T9N, L128P, R134K, G142R, G176D, T181P, R216H, D219N, R234H, P285A, P285T, R306H, N307I, T352I, P353A, P491R, A505V, R513H, S575P, W597C, A675T, P744S, C765G, and R776H; nonsense mutations include C133X, K378X, Q380X, E460X, R471X, Q540X, and W597X; frameshift mutations include W417fsX432, E455fsX493, V563fsX580, and L650fsX652; and splice site mutations include IVS11-2A/G, IVS11-7T/G, IVS11+1G/A, and IVS17+1delG as well as amino acid deletion mutation K212del and amino acid insertion mutation T319_N320insN [7, 22–25].

Congenital heterozygous hypoplasminogenemia is a rare condition and was first described in 1982 in a patient with thromboembolic disease [34]. It was earlier thought that heterozygous hypoplasminogenemia might be a risk factor for venous thrombosis; however, a study by Drew et al. has suggested that isolated heterozygous or homozygous/compound-heterozygous hypoplasminogenemia by itself is not a risk factor for deep venous thrombosis [35]. In 1997, Schuster et al. demonstrated distinct homozygous and compound-heterozygous mutations in the PLG gene and confirmed the autosomal-recessive inheritance of this disorder [23]. Studies have shown the incidence rate of hypoplasminogenemia worldwide to be less than 1%. A large epidemiologic study performed in blood donors in the United Kingdom demonstrated an observed rate of heterozygous (asymptomatic) plasminogen deficiency of 25 out of 9611 patients or 0.26% [36]. The incidence rate of (heterozygous) hypoplasminogenemia has been roughly estimated in different geographic regions at 0.35% (only in white subjects) in Minnesota, USA [37], at 0.13% in Southern Germany [38], and at 0.42% in Japan [39].

In our case, the GATK Best Practices framework was followed for the identification of variants in the sample. Gene annotation of the variants was performed using the VEP program against the Ensembl release 87 human gene model [40]. Clinically relevant mutations were annotated using published variants in the literature and a set of disease databases—ClinVar, OMIM, GWAS, HGMD, and SwissVar. Common variants were filtered based on allele frequency in 1000 Genomes phase 3, ExAC, EVS, dbSNP147, 1000 Japanese Genome, and our internal Indian population database. Nonsynonymous variant effects were calculated using multiple algorithms such as PolyPhen-2, SIFT, MutationTaster 2, MutationAssessor, and LRT. Only nonsynonymous and splice site variants found in the clinical exome panel consisting of 8332 genes were used for clinical interpretation. Silent variations that do not result in any change in amino acid in the coding region were not reported. A homozygous missense variation in exon 7 of the PLG gene (chr6:161137712G>A; depth: 50x) that results in the amino acid substitution of histidine for arginine at codon 235 (p.Arg23His; ENST00000308192.9) was detected. Inheritance was autosomal recessive. The observed variation lies in the kringle domain of the plasminogen protein and has previously been reported as pathogenic as per ClinVar and SwissVar [18–20].

The most characteristic features of this destructive periodontal disease are widespread membranous, nodular gingival enlargements in both maxilla and mandible leading to rapid tooth loss despite several treatment attempts [11]. Several therapeutic approaches to reduce the bacterial load to decrease the inflammation and thus the progression of the disease have been attempted; these procedures include scaling and root planing, chlorhexidine rinsing, administration of antibiotics, and periodontal surgery [9, 25, 30]. Neering et al. have reported that patients with PLG-deficiency Type I may benefit from nonsurgical periodontal therapy including full mouth disinfection in combination with adjunctive antibiotic therapy and a strict supportive periodontal therapy regime every three months [32]. Most of the reports have been described as failures due to rapid gingival regrowth and progressive bone loss [5]. Scully et al. suggested that gingival lesions can be controlled by topical heparin or intravenous purified plasminogen [41]. Gunhan et al. stated that systemic fibrinolytic and antithrombotic agents may prove more beneficial than local treatments, because the ligneous lesions tend to involve several mucosal areas [11]. Traditional periodontal treatment options for managing gingival enlargements include either gingivectomy or flap surgeries. The main objective is to excise the enlargements and reestablish healthy gingival margins and contours. The healing pattern in flap surgeries is by primary intention, whereas in gingivectomy, it involves healing by secondary intention. These healing mechanisms involve the formation of a fibrin clot, which creates a framework for normal periodontal tissues to grow back. However, in patients with plasminogen deficiency, the inadequacy of plasmin results in the overgrowth of fibrin. This is the main reason for the failure of conventional periodontal treatment modalities in these patients. A case report indicated that the treatment with warfarin exerts protection against relapsing gingival hyperplasia over an observation period of 3 years in a 54-year-old patient. The authors reported about a combination of gingivectomy, an administration of 20 mg doxycycline daily, and the use of a 0.12% chlorhexidine digluconate mouth rinse. One week after surgery, the patient started with 5 mg warfarin daily for an indefinite time [42]. Silva et al. reported a complete regression of oral mucous lesions after systemic and topical corticosteroids [43]. A recent clinical trial by Shapiro et al. was the first to evaluate the clinical utility of Glu-plasminogen in children and adults with congenital plasminogen deficiency. Human Glu-plasminogen replacement therapy was administered intravenously at 6.6 mg/kg every 2-4 days to 14 patients for a period of 12 weeks. Upon administration, results show the achievement of physiological levels of plasminogen activity that temporally coincided with clinical efficacy and improved disease management [44]. In the present case, the 26-year-old male patient reported with gingival enlargements present since childhood. Excision of the lesion was done with a surgical blade on one side and with a diode laser on the other side. However, recurrences of the lesions were seen within 3 months.

## 4. Conclusion

This case report discusses the diagnosis of ligneous periodontitis associated with Type 1 plasminogen deficiency in a 26-year-old male patient. The clinical presentation was that of a generalized gingival enlargement. Through this case report, we would like to stress on the importance of a thorough history taking, intraoral clinical evaluation, radiographic evaluation, and haematological examination, as a varied etiopathogenesis is reported for gingival enlargements. A histopathological examination of the biopsy specimen helped in confirming the diagnosis. Genetic testing helped in precisely identifying the gene, location, variant, zygosity, and inheritance pattern. Excision of the enlargements by means of surgical and laser techniques were not successful and recurrence occurred. The lack of efficacious therapy has increased the suffering of affected patients under the burden of this very rare blood disease.

## Abbreviations

LP: Ligneous periodontitis
PLG: Plasminogen
LC: Ligneous conjunctivitis
SGOT: Serum glutamic oxaloacetic transaminase
AST: Aspartate amino transferase
SGPT: Serum glutamic pyruvic transaminase
ALT: Alanine transaminase
PAS: Periodic acid-Schiff
BWA program: Burrows-Wheeler aligner program
SIFT: Scale invariant feature transform
OMIM: Online Mendelian Inheritance in Man
GATK: Genome Analysis Toolkit
VEP: Variant effect predictor
GWAS: Genome-wide association study
HGMD: Human Gene Mutation Database.
